# Non-tuberculous Mycobacterium Rhinosinusitis in an Immunocompetent Patient

**DOI:** 10.7759/cureus.44002

**Published:** 2023-08-23

**Authors:** Michelle G Tomlinson, Shreya Chidarala, Brian C Lobo, Gautam S Kalyatanda

**Affiliations:** 1 Infectious Diseases and Global Medicine, College of Medicine, University of Florida, Gainesville, USA; 2 Otolaryngology - Head and Neck Surgery, University of Florida, Gainesville, USA

**Keywords:** atypical infection, nasal mass, endoscopic sinus surgery (fess), mycobacterium abscessus, immunocompetent patients, chronic rhinosinusitis, nont-tuberculous mycobacteria

## Abstract

Non-tuberculous mycobacteria (NTM) are slow-growing opportunistic pathogens that cause a variety of cutaneous, soft tissue, and pulmonary infections. On rare occasions, NTM causes chronic rhinosinusitis, with the majority of cases presenting in immunocompromised individuals. Other potential risk factors include the presence of foreign bodies, previous sinus surgery or chemoradiation, and use of contaminated water in sinus rinses. We report here a rare case of NTM rhinosinusitis in an otherwise immunocompetent 66-year-old female. The patient underwent functional endoscopic sinus surgery where intraoperative acid-fast bacteria cultures grew *Mycobacterium abscessus.* She received five weeks of broad-spectrum IV antibiotic therapy followed by three months of oral azithromycin, tigecycline, and linezolid. A one-year post-operative visit showed appropriate healing without crusting or visible infection. This case contributes to the small handful of documented presentations of NTM rhinosinusitis in immunocompetent patients. NTM should be considered when patients present with refractory rhinosinusitis as they may require extended courses of antibiotic treatment. Familiarity with risk factors can further expedite making a diagnosis, ensuring prompt initiation of treatment and relief of symptoms for patients.

## Introduction

Chronic rhinosinusitis (CRS) is a common disease process affecting 5-12% of the global population. Due to the vague nature of defining CRS and overlapping comorbidities (i.e. allergic rhinitis), prevalence is likely overestimated [1]. CRS is an inflammatory disease of the paranasal sinuses and mucosal linings that last at least 12 weeks in duration. Two of four cardinal symptoms in adults are needed for diagnosis: 1) nasal mucopurulent drainage, 2) nasal obstruction, 3) facial pain or pressure, and 4) loss or reduction of olfaction [2]. *S. aureus* and anaerobic organisms (*Prevotella, Porphyromonas, Fusobacterium, and Peptostreptococcus spp*) are common bacterial isolates for CRS [3]. Refractory cases should raise concern for fungal etiologies, vasculitis (GPA, sarcoidosis), immunodeficiency, or poor mucociliary clearance. 

Non-tuberculous mycobacteria (NTM) are opportunistic, rapidly or slow-growing, acid-fast bacilli pervasively found in soil, water, and the natural environment. Pulmonary disease, superficial lymphadenitis, and soft tissue infections are localized clinical manifestations of NTM, though disseminated disease is seen in HIV and immunocompromised patients. Other risk factors for NTM isolates include prior chemoradiation and foreign body presence [4]. NTM sinusitis treatment is complicated by increased resistance to commonly used antimicrobial drugs. Thus, NTM sinusitis is a rare cause of rhinosinusitis with only a handful of cases presented in the past three decades. Here, we detail a case of NTM rhinosinusitis in an immunocompetent patient at our institution. 

## Case presentation

Patient one is a 66-year-old female with a past medical history notable for CRS, acquired hypothyroidism well controlled with 100mcg levothyroxine daily, blood transfusion, and turbinate hypertrophy that was treated with right sinus surgery 10 years prior to presentation. The procedure was uncomplicated and provided significant relief.

She recently presented to an external clinic complaining of a three-month history of chronic, persistent nasal obstruction, sinus and retro-orbital facial pressure, facial pain, colored mucopurulent drainage, headaches, aural fullness, and dizziness. The patient had just undergone a left upper root canal secondary and a recent tooth extraction, during which her tooth shattered and was not completely removed. While awaiting removal, she received multiple courses of antibiotics from her periodontist and developed throbbing, left periorbital pain. Her history is otherwise unremarkable and negative for tobacco, alcohol, or recreational drug use. She is a retired educator and previously worked in a microbiology lab and is devoid of exposure to acid-fast bacilli, fungi, and additional tuberculosis risk factors.

After referral to a local ENT, she was deemed refractory to multiple topical and oral antibiotics, steroids, antihistamine (azelastine), and high-volume nasal topical irrigations. She presented to our institution for a second opinion. On assessment, the patient was afebrile and in no acute distress (​​BP 146/79, HR 67, Temp 36.3°C). Her nasal obstruction (left > right) was positional and improved in an upright position and exacerbated by bending down. On physical exam, she was tender on percussion in the left frontal and maxillary sinus. The nasal septum was deviated to the left and no masses. The head and neck exam was otherwise unremarkable. Initial cultures were negative. Initial diagnostic nasal endoscopy demonstrated synechia at the right middle turbinate and lateral wall, and thick, yellow mucus in the maxillary sinus outflow tract. A maxillofacial CT scan demonstrated thickening of the left frontal, ethmoid, and maxillary sinuses, with the left side being more affected than the right. Maximal medical therapy was recommended at this time, and daily antihistamines, saline irrigation, and nasal steroids were initiated.

With unchanged symptoms after three months, functional endoscopic sinus surgery was planned. Intraoperatively bony osteitis, hypertrophy throughout the ethmoid cavity, and a 3mm bony dehiscence on the cranial wall of the right orbit were visualized. Bilateral and endoscopic total ethmoidectomy, frontal sinusotomy, maxillary antrostomy with removal of tissue, concha bullosa resection, and turbinate outfracture were done. Though extensive damage was present, the bone fragments in the pathology sample did not elicit osteomyelitis or pathologic changes. The patient was discharged afebrile and hemodynamically stable. Intraoperative cultures of the biopsy specimen eventually resulted in *Mycobacterium abscessus* without acid-fast bacilli visualized on direct smear. Culture sensitivities were susceptible to amikacin, cefoxitin, clarithromycin, and impinenem with tentative susceptibility to azithromycin, clofazimine, and tigecycline.

The patient then had a peripherally inserted central catheter line placed and antibiotics were. Plan was to complete a total of 12 months of antibiotic therapy with the first six to eight weeks being IV therapy (meropenem, tigecycline, clarithromycin PO). However, five weeks after initiation, all antibiotics were discontinued due to the presence of transaminitis and side effects of ocular migraines, tinnitus, and nausea. She was started on oral antibiotics with azithromycin, tigecycline, and linezolid. A second irrigation six weeks post-operatively at the ENT clinic only grew normal flora. Postoperative physical exam revealed midline septum and absence of previously noted crusting residual tenderness of sinuses. Post-op endoscopy after rigid nasal debridement demonstrated turbinates in the appropriate position and patent air spaces bilaterally. The patient completed three months of oral antibiotic therapy. A one-year post-operative visit showed appropriate healing without crusting or visible infection.

## Discussion

Nontuberculous mycobacteria are environmental opportunistic pathogens found ubiquitously in natural water and soils. Common species include* M. avium complex, M. chelonae, M. fortuitum, *and *M. abscessus *[5,6]. NTMs have been reported to cause pulmonary infections as well as cutaneous and soft tissue infections such as cervical lymphadenitis, abscesses, joint infection, and surgical site infection [7]. Rhinosinusitis is an uncommon manifestation of NTM’s. A recent epidemiology report in Minnesota estimated the disease burden of extrapulmonary NTM to be 1.8/100,000 people/year, with 8% of reported infections located in the sinus. The most common mycobacteria isolates included *M. chelonae, M. fortuitum, *and *M. abscessus *[5]. Another epidemiology report in North Carolina estimated an NTM infection annual prevalence of 13.7/100,000, including pulmonary and extrapulmonary infections. 13/195 reported extrapulmonary infections originated from the paranasal sinuses, with the most identified species including *M. abscessus complex* and *M. mucogenicum*. Of note, the prevalence of NTM extrapulmonary infection was higher in younger black patients, possibly due to higher rates of HIV or other immunocompromised states within this population [6].

A majority of the cases of NTM CRS in the literature occur in individuals with underlying HIV infection, cystic fibrosis, or other immunocompromising conditions [8-13]. Only a handful of cases have been reported in immunocompetent patients [14,15]. Several chart reviews have been conducted to identify common risk factors for NTM CRS. Suh et al. proposed previous endoscopic sinus surgery (ESS) as a risk factor for colonization and subsequent infection. Similarly, Solyar et al. and Tichenor et al. reported that most patients diagnosed with NTM CRS had a history of ESS. Both studies proposed sinus rinses using contaminated household water as a potential source of infection due to a noticeable correlation between NTM CRS diagnoses and active use of nasal irrigation for treating other sinus symptoms. Tichenor et al. further investigated this theory of nasal rinse inoculation by comparing NTM culture isolates from household water samples with patients’ sinus culture isolates. Rep-PCR fingerprinting and pulse field gel electrophoresis confirmed that 3/8 household water samples had *M. avium* isolates clonally related to the patient’s isolates.

NTM as an underlying cause of CRS is rare, and as such is difficult to identify. NTM should be suspected in patients with a long history of refractory CRS that has been unresponsive to standard therapies that are aimed toward the more common pathogens. Initial acid-fast bacterial (AFB) tissue staining is not sensitive or specific [12,16], as shown by our patient’s negative stain. NTM AFB cultures are standard for diagnosis but require two to eight weeks to grow, further delaying the identification of the pathogen and the appropriate treatment [7]. Additionally, due to the low prevalence of disease, obtaining AFB cultures as a standard workup for all CRS cases would not be cost-effective. Walsh et al.[17] proposed a more selective workup, only obtaining endoscopic-directed AFB cultures in cases of refractory CRS with a high index of suspicion for NTMs. 

Once a definitive diagnosis is established, treatment typically involves long-term antibiotic therapy [4,9,11,12,16-20], surgery [13], or some combination of the two [15]. Duration of antibiotic regimens varies considerably, ranging from one to 13 months. Further investigation would be required to determine the optimal length of antibiotic treatment. However, it would be difficult to provide evidence-based treatment recommendations due to the rarity of the disease as well as variability in presentation.

## Conclusions

In this case, we present an immunocompetent individual with a history of prior sinus surgery who was diagnosed with refractory NTM CRS, and treated with prolonged IV and oral antibiotics. This case adds to a growing body of literature documenting the clinical presentation of NTM CRS in immunocompetent patients. It is important that physicians are aware of these cases in order to promptly diagnose the condition and begin appropriate treatment. 
